# ^1^H NMR-Based Biochemometric Analysis of *Morus alba* Extracts toward a Multipotent Herbal Anti-Infective

**DOI:** 10.1021/acs.jnatprod.2c00481

**Published:** 2022-12-21

**Authors:** Julia Langeder, Kristin Döring, Hannes Schmietendorf, Ulrike Grienke, Michaela Schmidtke, Judith M. Rollinger

**Affiliations:** † Division of Pharmacognosy, Department of Pharmaceutical Sciences, University of Vienna, Josef-Holaubek-Platz 2, 1090 Vienna, Austria; ‡ Vienna Doctoral School of Pharmaceutical, Nutritional and Sport Sciences, University of Vienna, Josef-Holaubek-Platz 2, 1090 Vienna, Austria; § Section of Experimental Virology, Department of Medical Microbiology, Jena University Hospital, Hans-Knöll-Straße 2, 07745 Jena, Germany

## Abstract

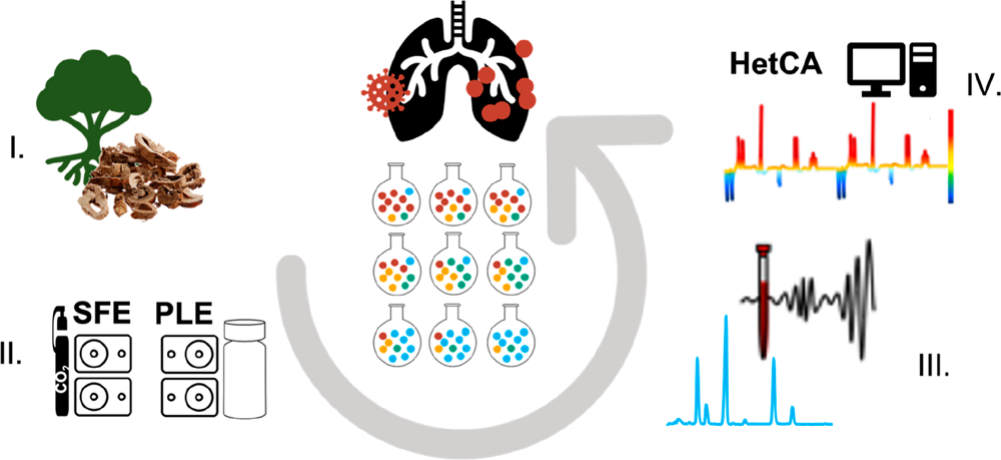

Mulberry Diels–Alder-type adducts (MDAAs) derived from the white mulberry tree were discovered recently as dual inhibitors of influenza viruses and pneumococci. For the development of a natural product based remedy for respiratory infections, the aim was to (i) identify the most prolific natural source of MDAAs, (ii) develop a protocol to maximize the content of MDAAs in *Morus alba* extracts, (iii) unravel constituents with the highest anti-infective potential within multicomponent mixtures, and (iv) select and characterize a hit extract as a candidate for further studies. Validated quantitative UPLC-PDA analysis of seven MDAAs (**1**–**7**) revealed the root bark as the best starting material and pressurized liquid extraction (PLE) as the optimum technique for extraction. Extracts enriched in MDAAs of a total content above 20% exerted a potent dual anti-influenza virus and antipneumococcal activity. For a detailed analysis of the most bioactive chemical features and molecules within the extracts, ^1^H NMR-based heterocovariance analysis (HetCA) was used. According to the multivariate statistical analysis procedure conducted, MDAAs exclusively accounted for the in vitro anti-influenza viral effect. The anti-infective profile of one hit extract (MA60) investigated showed a good tolerance by lung cells (A549, Calu-3) and pronounced in vitro activities against influenza viruses, *S. pneumoniae*, *S. aureus*, and inflammation.

Influenza A viruses are a major cause of acute respiratory infections, especially affecting the lower respiratory tract.^1,2^ Additional bacterial superinfections that are caused mainly by *Streptococcus pneumoniae* (pneumococci), *Staphylococcus aureus*, and *Haemophilus influenzae* aggravate the disease progression. These co-infections are involved in increased morbidity and mortality rates, leading to a significant impact on health care systems worldwide.^3^

Natural products have evolved over thousands of years under evolutionary pressure as a defense strategy against hostile organisms.^4,5^ Previous investigations of extracts, fractions, and constituents of the white mulberry tree (*Morus alba* L., Moraceae) revealed several bioactivities, e.g., inhibition of RNA and DNA viruses such as influenza A virus,^6^ dengue virus,^7^ Ebola virus,^8^ Herpes Simplex virus,^9^ and SARS-CoV-2.^10−13^ Mulberry Diels–Alder-type adducts (MDAAs), biosynthetically derived from [4+2]-cycloaddition of chalcones and dehydroprenylphenols, recently have been discovered to disrupt the lethal interplay of influenza viruses and pneumococci in vitro.^6,14^ In addition, anti-inflammatory activities were described for root bark extracts and constituents of the white mulberry tree that might help to control lung inflammation including bronchitis.^15−17^

By coevolution with a multitude of different microorganisms, plants produce an array of defense chemicals.^18^ Following Nature’s successful role model of the multicomponent defense strategy of plants, the objective of the present study was to identify a multipotent therapeutic agent able to combat respiratory infections of both viral and bacterial origin. However, further preclinical studies require a sufficiently bioactive and well-characterized material to avoid the challenging synthesis or the elaborate fractionation or even isolation of the complex MDAAs. Accordingly, the following steps were performed: (i) generation of extracts from different mulberry plant parts, (ii) determination of the quantitative composition of MDAAs, (iii) in vitro antiviral, antibacterial, and anti-inflammatory profiling, (iv) evaluation of the data complexity by a biochemometric tool to identify the anti-infective molecular features/molecules within the extracts, and (v) selection and further in vitro characterization of a hit extract candidate for future in vivo studies.

## Results and Discussion

**Figure 1:**
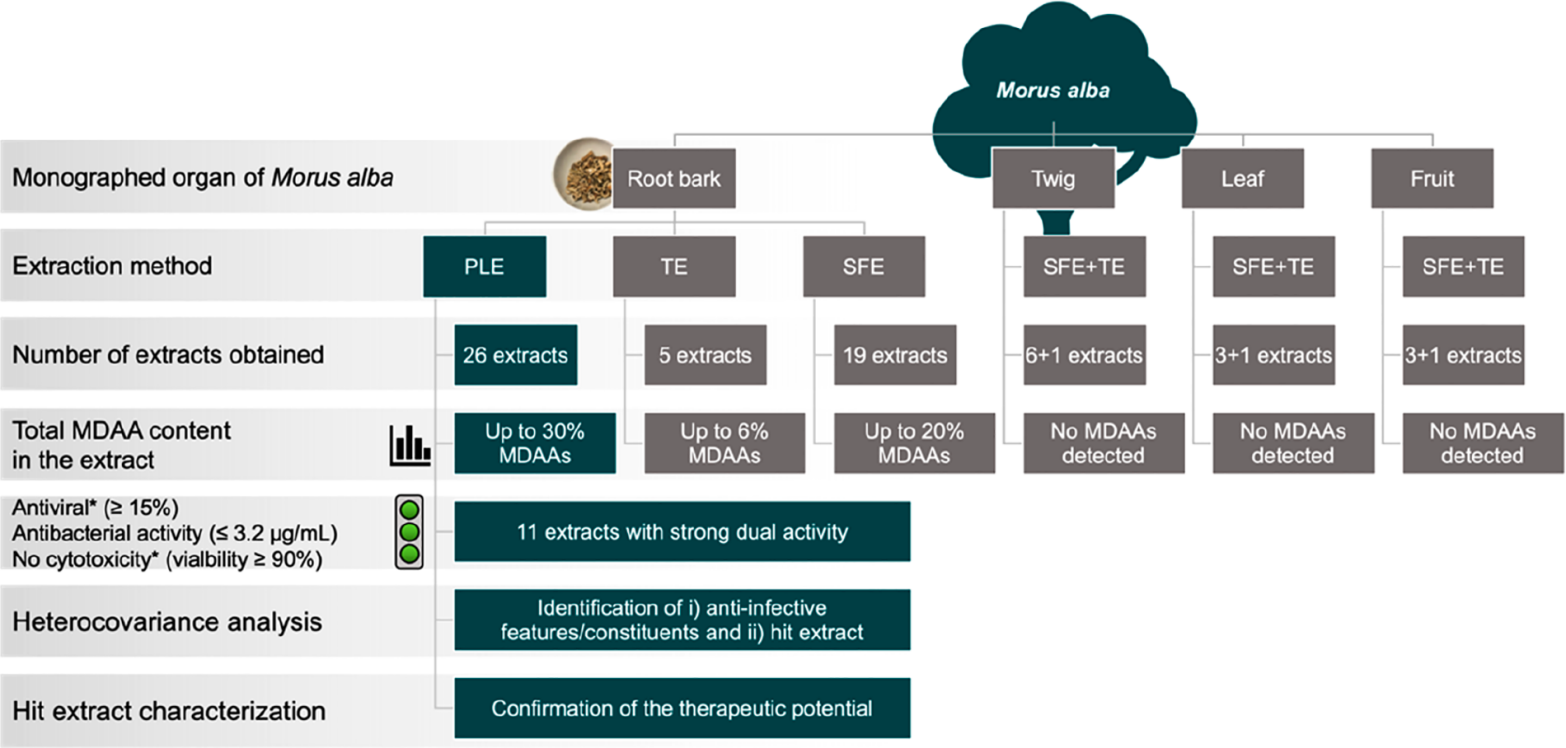
Flowchart describing the processing steps starting from four *M. alba* plant parts toward the hit extract selection (PLE, pressurized liquid extraction; SFE, supercritical fluid extraction; TE, traditional extraction; *tested at 10 μg/mL). Initially, all four plant parts were extracted with SFE and TE for biocompatibility reasons. As the root bark was the only plant part to contain MDAAs, PLE additionally was applied to further increase the total MDAA content.

Prenylated flavonoids bearing a densely functionalized hydroxy-benzofuro[3,2-*b*]chromenone ring system, the so-called sanggenons (i.e., MDAAs), isolated from the root bark of *M. alba*, have been reported to inhibit both influenza viruses and pneumococci in vitro.^6^ Hence, MDAAs were selected as marker compounds against respiratory infections. The aim of this study was to identify the most abundant plant source of MDAAs and to detect the most bioactive constituents. To avoid overlooking minor highly active compounds from a different or even the same structural class, heterocovariance analysis (HetCA)^19,20^ was applied as a biochemometric tool (Figure 1).

### Extract Preparation from Various Parts of the White Mulberry Tree

The *Chinese Pharmacopoeia* contains monographs for four different plant parts of *M. alba*, namely, the root bark, leaves, fruits, and twigs.^21^ Hence, with the aim of enriching MDAAs, the herbal material of all four plant parts was extracted using more specialized (e.g., supercritical fluid and pressurized liquid extraction) as well as traditional (e.g., maceration, decoction) extraction techniques. Supercritical fluid extraction (SFE) was chosen first to benefit from the applicability of biocompatible solvents such as ethanol and supercritical CO_2_. By leveraging various parameters like temperature, pressure, the ratio of CO_2_ and ethanol, or the extraction mode (static/dynamic), 29 supercritical fluid extracts (SFEs) were generated. Detailed information about the extraction parameters used is given in Table S1 (Supporting Information). Additionally, maceration with ethanol (96%) was performed with all four *M. alba* plant parts. Moreover, a decoction of the root bark was prepared in accordance with its application in Traditional Chinese Medicine. Further, a methanol extract (MA22) produced by ultrasonication, a 50% hydromethanol extract (MA23) obtained via reflux heating, and a 60% hydroethanol extract (MA21) were all prepared.

### Establishment of a Quantitation Method for MDAAs

For the quantitative analysis of MDAAs in the generated extracts, respective standard compounds were isolated from a dichloromethane-defatted methanol root bark extract by means of flash chromatography and UPLC. Altogether six constituents were obtained in high purities (according to UPLC-ELSD) and sufficient quantities: kuwanon L (**1**: 93%, 4.7 mg), sanggenon D (**2**: 99%, 730 mg), sanggenon G (**4**: 98%, 10.8 mg), sanggenon O (**5**: 99%, 5.25 mg), sanggenon E (**6**: 95%, 6.6 mg), and sanggenon C (**7**: 99%, 515 mg). Compound **3** was identified tentatively as sanggenon B^6^ and quantitated by comparing the peak area with that of congener **7**. The structures of the isolated MDAAs were confirmed by means of LC-ESIMS and 1D and 2D NMR spectra in comparison to published literature for **1**,^22^**2**,^23^**4**,^24^**5**,^25^**6**,^26^ and **7**.^27^

**Chart 1:**
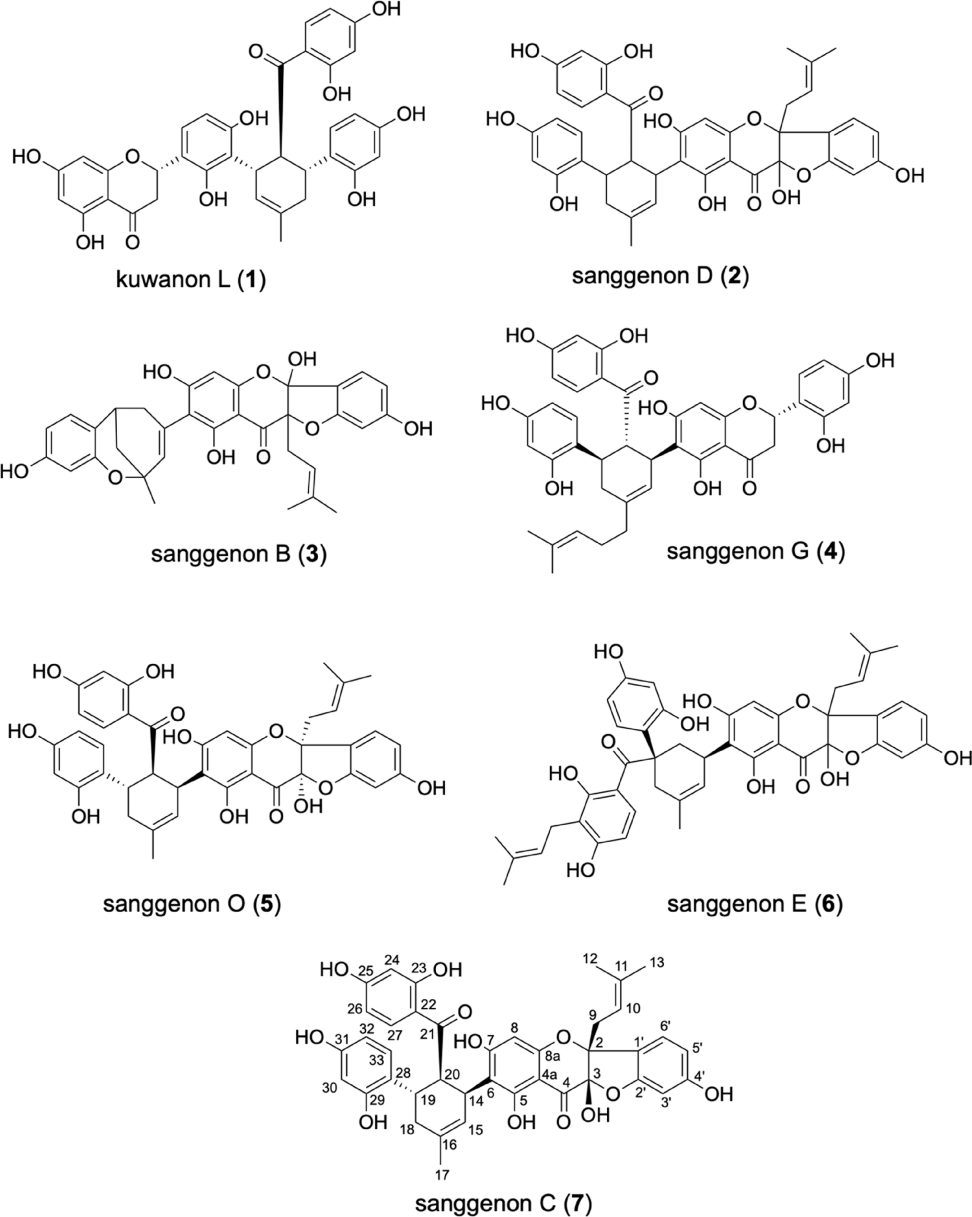


Compounds **2** and **5**–**7** are characterized by a *cis*-3-hydroxy-2-prenylflavanone core and an ether linkage between C-2′ and C-3, forming a 5,6-fused ring system. They contain also a tetrasubstituted cyclohexene ring and are so-called Diels–Alder-type adducts with a highly oxygenated 2′-hydroxychalcone unit. After the successful development of a suitable chromatographic method using a BEH Phenyl column (2.1 × 100 mm, particle size 1.7 μm) on a Waters Acquity H-class UPLC system, compounds **1**–**7** were quantitated in the extracts generated using PDA detection at 205 nm. The identification of all seven compounds was based on the retention time in the PDA chromatograms compared to their standards. Quantitative measurements were validated in accordance with ICH guidelines^28^ (Table S1, Supporting Information). Good linearities were achieved for the investigated compounds **1**, **2**, and **4**–**7** with coefficients of determination (*R*^2^) above 0.9991. Limit of detection (LOD) and limit of quantitation (LOQ) levels were determined using 1 mL of methanol spiked with the respective standards diluted to a final concentration of approximately 60 ng/mL, respectively. Values varied from 6.67 to 102 ng/mL for LOD and from 22.2 to 340 ng/mL for LOQ. Intraday and interday precision of the proposed method was expressed in terms of standard deviation and ranged from 0.9% to 10.1%. Furthermore, accuracy was determined in spiking experiments (high, medium, and low) with standard compound **7**, resulting in a recovery rate of 84.2% to 95.8%. Dried extracts were prepared according to established protocols and analyzed in triplicate. The compiled quantitative results are listed in Table S2 (Supporting Information).

### Selection of the Most Suitable White Mulberry Plant Parts

None of the seven selected marker compounds was detected in either the SFE or the traditional extracts of white mulberry leaves, fruits, and twigs (data not shown). Only the root bark was enriched in bioactive MDAAs. Hence, *M. alba* root bark was selected as the starting material for further extract optimization steps. Depending on the applied extraction parameters, SFEs of the root bark showed a significant variance in their total content of MDAAs (Table S3, Supporting Information). Generally, when the herbal material is defatted with 100% CO_2_ (polarity similar to *n*-hexane), depending on the subsequent extraction step, the total content of MDAAs in the final extract can be increased significantly up to 20%. The extracts MA10, MA17, and MA18, with a total MDAA content of 17%, 20%, and 15%, respectively, were defatted with 100% CO_2_ in the dynamic mode with or without a static extraction step of 15 min. The second extraction step was carried out with 70% CO_2_ and 30% ethanol with a pressure between 200 and 300 bar and an oven temperature of 40 to 50 °C. In contrast, MA01 was generated without a defatting step using 70% CO_2_ and 30% ethanol. This procedure resulted in only a 4% total MDAA content. No MDAAs were detected in extracts obtained by 100% CO_2_ only (MA07). The analysis of extracts derived from more classical extraction methods resulted in a distinctly lower content of MDAAs (around 5%, MA20–MA24).

### Pressurized Liquid Extraction of Mulberry Root Bark

To investigate whether the content of MDAAs from the root bark can be further increased, pressurized liquid extraction (PLE) was used. Similar to SFE, PLE allows for a defatting step, but it has the advantage of being able to extract the defatted material afterward with a broad range of solvents of different polarity and thus to enrich specifically the targeted compound class. Settings for the 26 generated PLE extracts are given in Table S4 (Supporting Information).

Quantitative UPLC analyses revealed an efficient depletion of nonpolar constituents when the root bark was defatted with either petroleum ether or *n*-hexane. A notable enrichment of total MDAAs up to 32% was achieved by the subsequent extraction with mixtures of isopropanol and petroleum ether (1:1; 1:2; 2:1), resulting in extracts MA57 (25%), MA59 (31%), MA60 (29%), and MA61 (32%) (Table S5, Supporting Information). Extracts with a content of >20% MDAAs were prepared with isopropanol (MA50, MA54–MA56), acetone (MA44), acetonitrile (MA51), or a 1:1 mixture of petroleum ether and 1-propanol (MA63).

Thus, when comparing classical extraction methods (total MDAA content reaches approximately 2.5–5%) to more specialized techniques like SFE and PLE, the latter allowed for a six- to 11-fold increase in the presence of MDAAs (Figure 1).

### Identification of Most Potent, Dual-Acting PLE Root Bark Extracts

**Figure 2:**
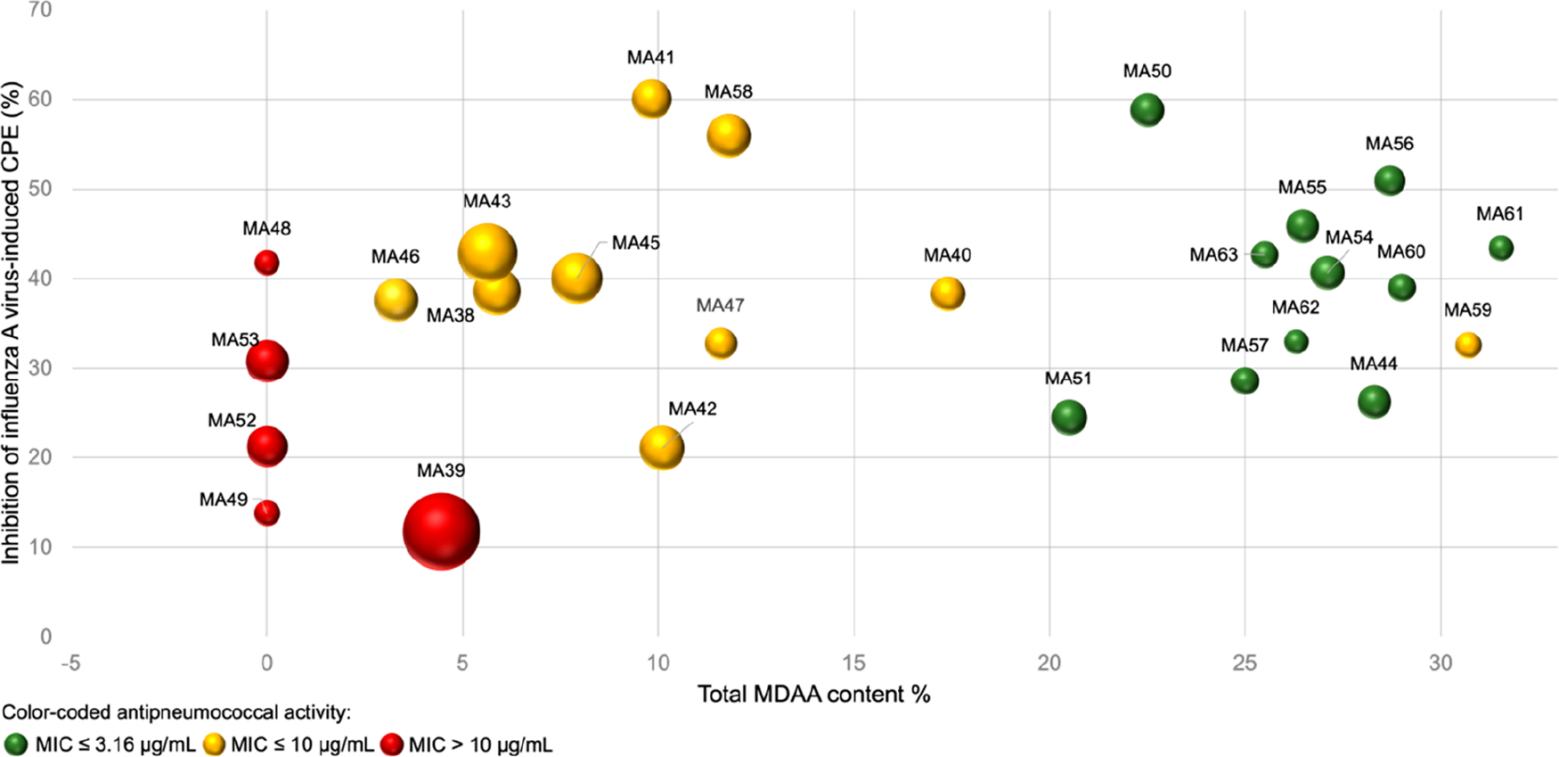
Inhibition (%) of influenza A virus-induced CPE by the 26 PLE extracts at 10 μg/mL plotted against the MDAA content (%). The anti-*S. pneumoniae* activity is color-coded, and the extraction yields are represented by the size of the spheres shown.

According to the quantitative analyses performed, the PLE root bark extracts produced showed the highest content of MDAAs wherein the composition of the quantitated marker compounds **1**–**7** varied (Table S5, Supporting Information; MA38–MA63). To identify the most potent dual-acting PLE root bark extracts, their anti-influenza virus activity at 10 μg/mL and their minimal inhibitory concentration (MIC) against *S. pneumoniae* were determined. The anti-influenza virus and anti-*S. pneumoniae* activities of the previously described MDAA-enriched fraction MAF^6^ served as a reference. With the exception of MA42, all extracts were well tolerated by MDCK cells at the concentration used (viability >90%; results not shown). In total, 11 extracts inhibited the influenza virus-induced cytopathic effect (CPE) more potently than MAF (≥15%) and exerted an equal or stronger antipneumococcal activity than MAF (MIC ≤ 3.12 μg/mL) (Figure 2). These 11 root bark extracts were characterized by an MDAA content of >20% (Table S5, Supporting Information). Further, the extract yield, which is important when considering the quantities needed for preclinical studies, was represented in each case by the size of the sphere ranging from the smallest yield of 0.76% (MA62) to the highest of 8.82% (MA39) (Figure 2).

### Anti-Infective Chemical Features Identified by ^1^H NMR-Based Biochemometry

In contrast to classical metabolomics, the field of biochemometry accumulates both chemical and biological data sets and is particularly suitable for drug discovery from natural sources. Previously, an efficient workflow to unravel active and inactive compounds in a multicomponent mixture prior to isolation was developed. This biochemometric system, named ELINA (Eliciting Nature’s Activities),^20,29^ included the following steps: (i) microfractionation of a bioactive extract; (ii) bioactivity testing and recording of ^1^H NMR and LC-ESIMS data for all microfractions; (iii) correlation of bioactivity data and chemical data with multivariate statistical tools to distinguish active from inactive compounds. One of the statistical tools used is heterocovariance analysis,^19^ a technique to visualize the covariance and the correlation between ^1^H NMR resonances and bioactivity data resulting in color-coded pseudospectra (HetCA plots).

In the present study, as opposed to the classical ELINA workflow, no microfractionation of bioactive extracts was performed. In contrast, herein the use of different extraction parameters ensured a quantitative variance of constituents in the extracts generated that were needed for the subsequent statistical correlation of ^1^H NMR spectra with activity data. With this procedure, the aim was to answer the following questions: (i) apart from MDAAs, was there any other (minor) bioactive compound class present in the influenza A virus inhibiting extracts, and (ii) could the biochemometric HetCA analysis reveal differences within the structural class of MDAAs regarding the correlation with activity, to discern if some MDAAs are more potent in activity than others?

Aliquots of the 26 PLE extracts were subjected to ^1^H NMR measurements. All spectra were acquired at a concentration of 3 mg/mL using the same conditions to obtain an identical signal-to-noise ratio. Proton resonance signals are proportional to their molar concentration allowing for a direct comparison of these 26 extracts with the determined percentage of inhibition of influenza virus-induced CPE. As an output of this analysis, a ^1^H NMR pseudospectrum was obtained showing positively correlated resonances in red (facing upward) and negatively correlated ones in blue (facing downward).

**Figure 3:**
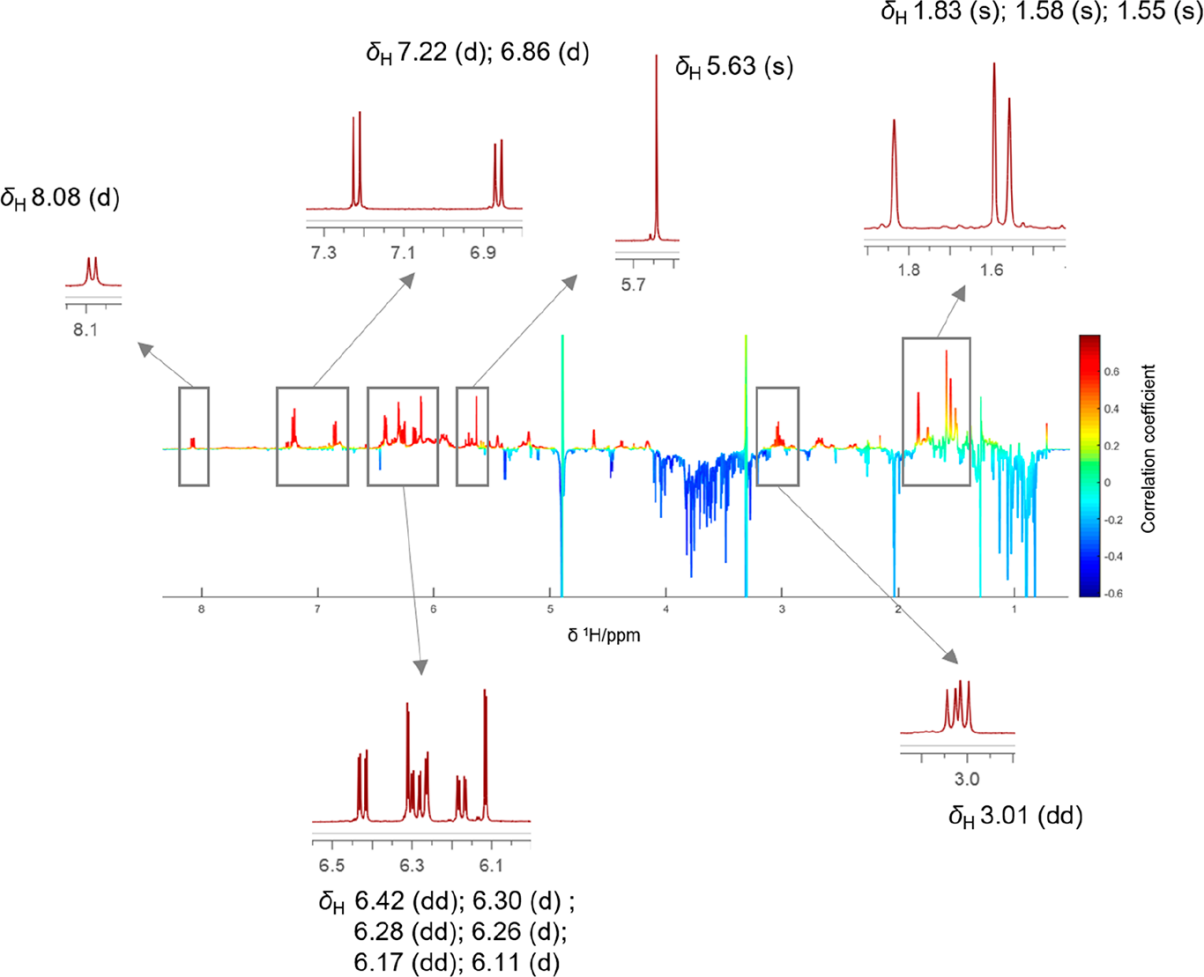
^1^H NMR pseudospectrum showing the heterocovariance (HetCA) of ^1^H NMR spectra and influenza A virus inhibition data of 26 extracts produced by PLE. The color code is based on the correlation coefficient: blue = negatively correlated with activity; red = positively correlated with activity. Red signals are compared to expansions of the ^1^H NMR spectrum of compound **7**.

To answer the initial research questions, the positively correlated resonances depicted in red in the HetCA pseudospectrum (between δ_H_ 5–8.5 and around δ_H_ 3 and δ_H_ 1.4–2) were compared to the ^1^H NMR spectra of individual MDAAs (**1**–**7**). This allowed for the individual assignment of resonances, particularly for compound **7** (Figure 3). The aromatic proton at C-27 was observed as a doublet at δ_H_ 8.08. Doublets at δ_H_ 7.22 and δ_H_ 6.86 were assigned to the protons at C-6′ and C-33, respectively. Further, three doublets at δ_H_ 6.30, δ_H_ 6.26, and δ_H_ 6.11 reflected protons at C-3′, C-30, and C-24. The three doublets of doublets at δ_H_ 6.42, δ_H_ 6.28, and δ_H_ 6.16 were assigned to protons at C-5′, C-26, and C-32, while the singlet at δ_H_ 5.63 resulted from the proton at C-8. The three singlets in the aliphatic region at δ_H_ 1.83, δ_H_ 1.58, and δ_H_ 1.55 belonged to the methyl protons at C-17, C-13, and C-12 (Figure 3).

Although the red signals between δ_H_ 5.60 and 7.50 might also have matched with the spectrum of compound **5** (Figure S1, Supporting Information), the remaining positively correlated resonances only fit its stereoisomer compound **7**.

Combining the information gained from HetCA analysis, the quantitative composition of MDAAs (Table S5, Supporting Information), and the dual inhibitory potency against influenza A viruses and pneumococci (Figure 3), it could be concluded (i) that there is no other (minor) bioactive compound class present in the bioactive extracts and (ii) that compound **7** is the major contributor to the anti-influenza activity observed.

### Tolerance by Lung Epithelial Cells and Anti-Influenza Virus, Antibacterial, and Anti-Inflammatory Potential of the Active Extract MA60

Aiming to gain greater insight into the therapeutic potential of the *M. alba* root bark extract for acute respiratory infections, the biological activities of one of the hit extracts, namely, MA60, were characterized in more detail. For comparison, the MDAA-enriched fraction MAF as well as the isolated MDAAs **4** and **7** (both showing dual activities as pure compounds^6^) were included in this investigation. In addition, the compatibility and antibacterial potential were evaluated of MDAA **2**, which despite being inactive against influenza A virus, represented the second major component of the *M. alba* root bark extract.

The effects of MAF, MA60, and compounds **2**, **4**, and **7** toward lung epithelial cells were evaluated by analyzing their influence on NADH activity and on the viability of A549 and Calu-3 cells when compared to an untreated control. As summarized in Table S6 (Supporting Information), all samples were well tolerated by both lung epithelial cell lines.

**Figure 4:**
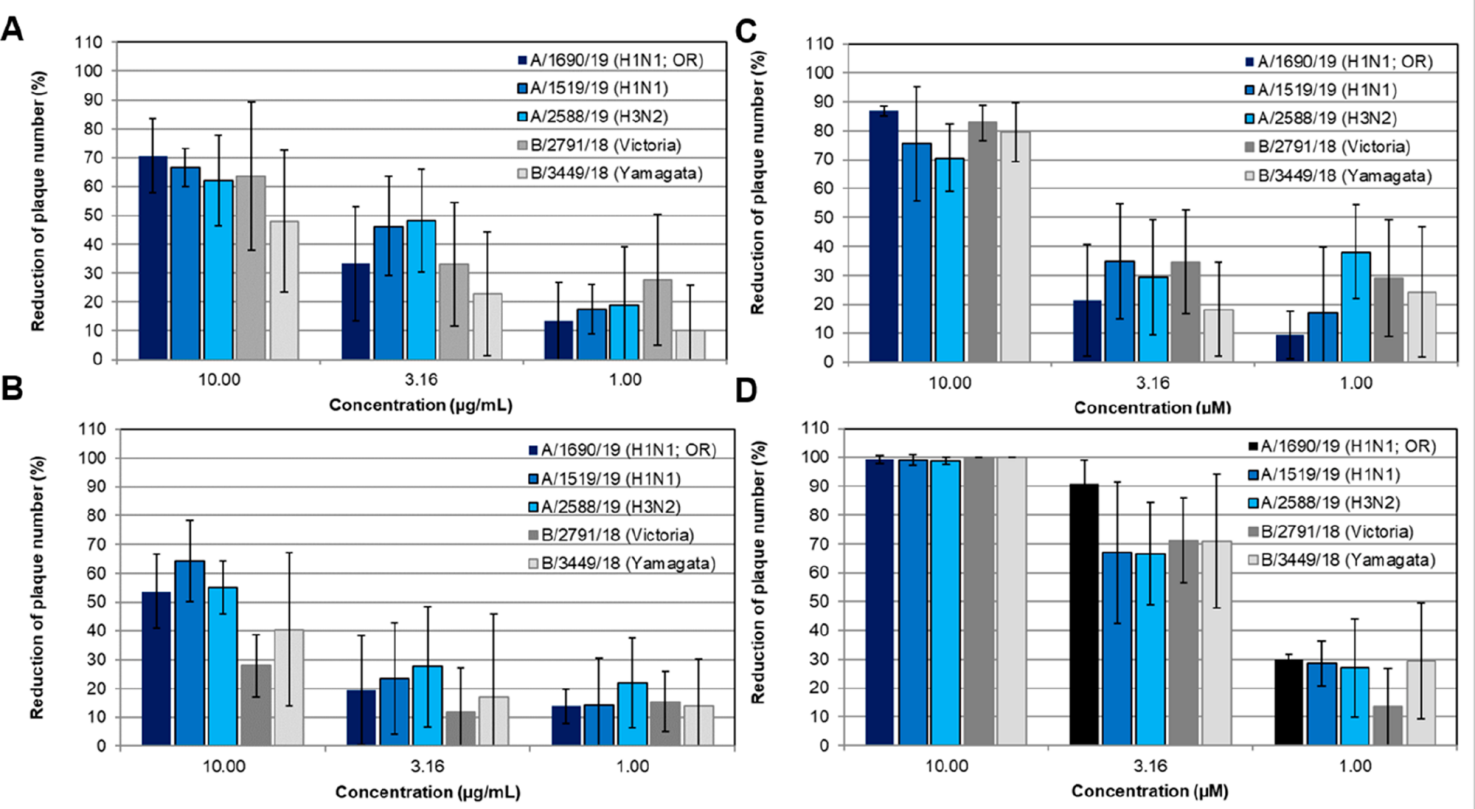
MAF (A), MA60 (B), and compounds **4** (C) and **7** (D) exerted a broad-spectrum anti-influenza virus activity. Antiviral activities are given for influenza A virus isolates of subtype A(H1N1)pdm09 (oseltamivir-resistant (OR) strain A/1690/19 and A/1519/19) and H3N2 (A/2588/19) as well as for influenza B virus isolates of both circulating genetic lineages Victoria and Yamagata (B/2791/18 and B/3449/18, respectively). MDCK cell monolayers were inoculated with suspensions of these five influenza A and B virus isolates with or without the indicated concentrations of test samples (in triplicate) at 37 °C for 1 h. After aspirating the inoculum, the test medium containing 0.4% agar and the indicated concentrations of MAF, MA60, and compounds **4** and **7** were added for 72 h.

To study the spectrum of anti-influenza virus activity of MA60, a recently described panel of three influenza A viruses and two influenza B viruses was applied.^30^ Based on their proven activity against an influenza virus A(H1N1)pdm09 isolate,^6^ MAF as well as compounds **4** and **7** were included for comparison in these studies. Plaque reduction assays were performed with the influenza virus panel and three noncytotoxic concentrations of the extracts and compounds in MDCK cells. The dose-dependent inhibition of plaque reduction is summarized in Figure 4. The results confirmed a broad anti-influenza virus activity of MA60, MAF, and compounds **4** and **7** also against influenza A and B viruses resistant to ion-channel blockers and/or the neuraminidase inhibitor oseltamivir.

**Figure 5:**
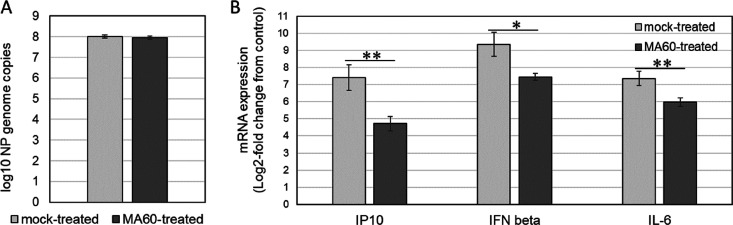
MA60 inhibits influenza A virus-induced cytokine induction in Calu-3 cells. Confluent Calu-3 cells were mock-treated with test medium or MA60-treated (10 μg/mL) and mock-infected or infected with influenza virus at MOI 1 for 24 h (all in triplicate). Results of qPCR with (A) a viral NP RNA-specific primer pair or (B) GAPDH-, IP10-, interleukin 6 (IL-6)-, and interferon β (IFN-β)-mRNA-specific primer pairs are shown. *p < 0.05, **p < 0.01 in an unpaired two-sided Student’s *t* test.

Besides the cytopathic effect of influenza virus infection, the damage from the immune response contributes to symptoms of influenza.^31^ Regarding further preclinical or clinical investigations, a reduction of inflammatory processes therefore might be an advantage. Based on the existing knowledge of the anti-inflammatory activities of *M. alba*(15−17) and the essential role of the bronchial epithelium for influenza virus infection, the effect of MA60 on influenza virus-induced interferon β (IFN-β), IP10, and interleukin 6 (IL-6) mRNA induction was determined in Calu-3 cells. As shown in Figure 5A and Figure S2A (Supporting Information), treatment with 10 μg/mL of MA60 did not inhibit influenza virus replication in Calu-3 cells. Results from the preceding work revealed viral neuraminidase as the main target of *M. alba* root bark extract and its active constituents.^6^ The inhibition of viral neuraminidase was confirmed for MA60 in a cell-based assay in the present study (Figure S3, Supporting Information). The activity of neuraminidase inhibitors in cell-based assays is known to be dependent on a balanced function of the viral hemagglutinin and neuraminidase.^32^ This criterion was fulfilled herein. However, the MA60 treatment reduced significantly the IP10, IL-6, and IFN-β response to the virus infection (Figure 5B and Figure S2B, Supporting Information). These results indicated the anti-inflammatory potential of *M. alba* root bark extract in influenza virus-infected bronchial cells.

Based on the known lethal synergism of influenza viruses with *S. pneumoniae* and *S. aureus*,^3^ also the antibacterial potential of MA60 was characterized in more detail. Broth microdilution assays were performed with two *S. pneumoniae* (DSM20566 and D39) as well as two *S. aureus* strains (ATCC43000 and ATCC25923) to determine the minimal inhibitory concentration (MIC) for MAF and compounds **2**, **4**, and **7**. The antibiotics ampicillin and rifampicin served as references for this work. Except for the ampicillin-resistant ATCC25923, the test bacteria were rifampicin- and ampicillin-sensitive (Table 1). The MDAA-enriched active extract MA60 inhibited the growth of both *S. pneumoniae* strains at 3.16 μg/mL as did the MDAA-enriched fraction MAF (Table 1). *S. aureus* was also highly susceptible to MA60, MAF, and the three MDAAs (Table 1). Thus, MA60 targets two major bacterial pathogens known to aggravate the course of influenza. The MIC values of compounds **2**, **4**, and **7** ranged from 1.7 to 3.16 μM and 3.16 to 10 μM for the *S. pneumoniae* and *S. aureus* strains, respectively (Table 1). The most potent activity in vitro was observed for compound **7**. These results indicated a contribution of all compounds tested to the antibacterial activity of MA60.

**Table 1:** Growth Inhibition of *S. pneumoniae* and *S. aureus* of the Active Extract MA60, MAF, and Compounds **2**, **4**, and **7**

		MIC^a^ [μM; multicomponent mixtures in μg/mL]			
		*S. pneumoniae*		*S. aureus*	
sample code		DSM20566	D39	ATCC43000	ATCC25923
multicomponent mixtures	MAF	3.16	3.16	1.00	0.77
	MA60	3.16	3.16	1.00	1.00
natural product isolates	**2**	3.16	3.16	10.00	10.00
	**4**	3.16	3.16	10.00	10.00
	**7**	2.08	1.72	3.16	3.16
antibiotics	ampicillin	0.02	0.01	15.40	1.00
	rifampicin	0.01	0.02	0.003	0.003

a Bacterial suspensions were grown with or without half-logarithmic dilutions of the test samples (in duplicate) at 37 °C overnight. The results represent the mean minimal inhibitory concentration (MIC) from three assays.

In conclusion, the root bark is the plant part of *M. alba* that is the most enriched in bioactive MDAAs relative to the other parts investigated, i.e., the leaves, fruits, and twigs. In comparing the efficiency of PLE to SFE and more traditional extraction methods, the PLE extraction procedure (using as a first step: defatting, and second extraction step with 2-propanol–petroleum ether (2:1 or 1:2) at 80 °C in flow mode including a static cycle of 5 min) is superior. This is highlighted not only by the analytical results obtained but also by the bioactive profile of MDAA-enriched extracts against the influenza A virus and *S. pneumoniae* and *S. aureus* strains investigated. By applying HetCA as a biochemometric tool on various extracts of the herbal drug investigated, compound **7** was identified as the main contributor to the observed anti-influenza virus activity. Based on these findings, MA60 was rated as one of the most promising multipotent active extracts and was therefore subjected to further in-depth investigations concerning its tolerance by lung cells and its antiviral, antibacterial, and anti-inflammatory potential in vitro. Compound **7**, one of the major constituents, was included in these studies and might serve as a potential marker compound for extract standardization in future studies. Accordingly, the results presented form a scientific rationale for relevant further preclinical in vitro and in vivo studies.

## Experimental Section

### General Experimental Procedures

UHPLC-ESIMS analysis was performed on a Dionex UltiMate 3000 UHPLC system (Thermo Fisher Scientific, Waltham, MA, USA) equipped with a Waters Acquity BEH phenyl column (2.1 × 100 mm, 1.7 μm) coupled to an LTQ XL linear ion trap mass spectrometer (Thermo Fisher Scientific). NMR experiments were performed on a Bruker Avance 500 NMR spectrometer (UltraShield) (Bruker, Billerica, MA, USA) with a 5 mm probe (TCI Prodigy CryoProbe, 5 mm, triple resonance inverse detection probe head) with *z*-axis gradients and an automatic tuning and matching accessory (Bruker BioSpin). The resonance frequency for ^1^H NMR was 500.13 MHz. The samples were measured at 298 K in fully deuterated methanol referenced to the residual nondeuterated solvent signal.

The quantitative analysis was performed on a Waters Acquity H-class UPLC system that consisted of a quaternary solvent manager equipped with an automatic sample manager (8 °C), a PDA, and an evaporative light scattering detector (ELSD). Compounds were separated using a mobile phase consisting of water (solvent A) and acetonitrile (solvent B) delivered at a flow rate of 0.3 mL/min through an Acquity BEH Phenyl column (dimensions 2.1 × 100 mm, 1.7 μm) kept at 40 °C. The gradient program [*T*(min)/%B] was 0/5, 1/50, 6.9/50, 7/98, 8.9/98, 9/5, and 10/5 with a total run time of 10 min, followed by an equilibration time of 3 min. The detection wavelength was set to 205 nm, and the injection volume was 5.0 μL. Subsequent data acquisition and processing were conducted by Waters Empower 3 software. The difference in molecular weight of compounds **3** and **7** was considered by multiplication of the peak area of **3** with the correction factor 0.805. To further purify compounds **4** and **5**, semipreparative UPLC was chosen for both purifications. The system was equally equipped as described before, but the ELSD was replaced by a fraction manager. For the sample preparation a total amount of 15 mg and 25 mg of fractions containing **4** and **5**, respectively, were dissolved in MeOH. Both compounds were separated in a 15 min gradient program with water (solvent A) and acetonitrile (solvent B), respectively. The gradient elution [*T*(min)/%B] for compound **4** was 0/5, 1/45, 8.9/50, 9/98, 11.5/98, 11.6/5, and 15/5 and was slightly adapted for compound **5** to 0/5, 1/50, 8.9/50, 9/98, 11.5/98, 11.6/5, and 15/5. The flow rate was maintained at 0.3 mL/min and the injection volume was 7 μL for **4** and 10 μL for **5**.

The methodical approach and the preparation of standards for quantitation were validated in accordance with ICH guidelines.^28^ In detail, stock solutions of all six standards were prepared by dissolving an accurately weighted amount of 1 mg/mL (level 0) in 1 mL of methanol. Nine further calibration levels (level 1 to level 9) were serially diluted in a ratio of 1:3 with the same solvent. Each level was injected in triplicate, and calibration curves were derived by integration of the peaks using Empower 3 software. For quantitation, 0.5–1.5 mg of extract was transferred into a 1.5 mL Eppendorf reaction vessel and dissolved in 1 mL of methanol. The solutions were mixed, ultrasonicated for 1 min, centrifuged at 13 500 rpm for 5 min, transferred into HPLC vials, and stored at 8 °C until analysis.

Antiviral and antibacterial assays were performed in biosafety level 2 laboratories.

### Plant Material

The *M. alba* root bark, leaves, fruits, and twigs (batch nos. 460797, 710723, 72042, and 570844) were purchased from Plantasia GmbH (Oberndorf/Salzburg, Austria) in 2018. Voucher specimens for the root bark (2018: JR-20190928-A1), leaves (JR-20190928-A3), fruits (JR-20190928-A4), and twigs (JR-20190928-A2) are deposited at the Department of Pharmaceutical Sciences, Division of Pharmacognosy, University of Vienna, Austria.

### Extraction and Isolation

Supercritical fluid extractions were performed on a Waters MV-10 SFE instrument equipped with a fluid delivery module, a 10-vessel extraction oven, a heat exchanger, an automated back pressure regulator, and an extraction collector with a makeup pump. ChromScope 1.6 software and 5 mL extraction vessels were used. Approximately 1 g of ground plant material was subjected to extraction. The following parameters were leveraged: oven temperature, pressure, flow rate, the composition of extraction solvent (% CO_2_ and % ethanol), duration of the first and second dynamic/static step, and number of applied samples. Detailed parameters are given in Table S3 (Supporting Information). In terms of extract optimization, only one parameter was changed for each extract to understand the effects of each setting. Several published studies report the successful usage of polar solvents in sub/supercritical fluid chromatography for natural products.^33−35^

Pressurized liquid extraction was performed on a Dionex Accelerated Solvent Extraction 350 obtained from Thermo Fisher Scientific. Approximately 1 g of ground plant material was transferred into 5 mL extraction cells. The exact same procedure as for the hit extract MA60 was repeated for approximately 15 g of root bark (34 mL extraction vessel). Details of the PLE setting for extract optimization are given in Table S4 (Supporting Information).

Ultrasonic extracts of all four plant parts were prepared by soaking 1 g of plant material with 30 mL of ethanol followed by 30 min of ultrasonication (Table S2, Supporting Information). A decoction (MA24) was prepared using 10 g of *M. alba* root bark soaked in cold water for 1 h followed by decoction for 20 min. After filtration through cotton wool, the filtrate was lyophilized. Additionally, two further extracts were generated using methanol as extraction solvent. For the first methanol extract (MA22), around 5.5 g of plant material was sonicated with 250 mL of MeOH for 15 min. The mixture was macerated for 18.5 h, then filtered through a cellulose filter under vacuum. After this step, again 4.7 g of ground plant material was weighed, but this time heated under reflux with 50% aqueous methanol (MA23) for 15 min in a water bath (80 °C).

For the ethanol extract (MA21), approximately 30 g of ground plant material was shaken with 500 mL of 60% aqueous ethanol for 3 days at 100 rpm at room temperature.

Methanol was of HPLC-grade and was used as an extraction solvent as well as for the preparation of all samples. Double-distilled water and HPLC-grade acetonitrile were used for the chromatographic separation. Solvents utilized for the preparation of different extracts for quantitation were distilled according to the *Austrian Pharmacopoeia* and included ethanol 60%, *n*-hexane, and petroleum ether. Isopropanol of analytical grade was used.

For large-scale isolation purposes, a methanol extract (MAM) was prepared. Approximately 2 kg of ground plant material was mixed with 7 L of CH_2_Cl_2_ for defatting at room temperature. After removing CH_2_Cl_2_, 3 L of MeOH was added for further maceration for 7 days. This was repeated two more times, and, after removing the solvent, 61 g of crude extract was obtained. Flash chromatography (FC) was chosen for the separation of the crude extract. All FC runs were executed on an Interchim puriFlash 4250 system with a PDA detector (200–600 nm) and an ELSD. For the initial fractionation, the crude extract (MAM) was applied in portions of 14 g in dry load operation. Conditions used for the fractionation are reported in Table S7 (Supporting Information). After the automated collection of the fractions, tubes with identical content were combined and the resulting fractions were coded MAM01_01 to MAM01_11.

For the preparative separation of MAM01_04, MAM01_05, MAM01_06, MAF, and MA60, direct injection was used by dissolving the respective sample in 2 mL of MeOH. The columns, flow rates, and gradient elution procedures used are reported in Table S7 (Supporting Information).

Fraction MAM01_04 was separated further by flash chromatography on a preparative GEMINI-NX C_18_ (250 × 10 mm) HPLC column, obtaining compound **6** (6.6 mg). Separation of MAM01_05 with flash chromatography yielded pure compound **7** (515 mg). Additionally, compound **5** was enriched in this fraction and isolated, but had to be further purified by UPLC, which yielded 5.25 mg of **5**. Compound **2** (730 mg) was isolated from fraction MAM01_06 by FC. Compound **4** was isolated from a previously reported fraction MAF^6^ using FC. The impurities present in this fraction were removed with UPLC to obtain compound **4** (10.8 mg) in high purity. For the isolation of compound **1**, the PLE extract (MA60LS) was separated using FC and led to 4.7 mg of compound **1**.

Thus, six substances were isolated: kuwanon L (**1**^22^), sanggenon D (**2**^23^), sanggenon G (**4**^24^), sanggenon O (**5**^25^), sanggenon E (**6**^26^), and sanggenon C (**7**^27^). Identification of isolated compounds was performed by interpretation of 1D and 2D NMR spectra and UHPLC-ESIMS. Purities were determined using an ultraperformance liquid chromatography–evaporative light scattering detector (UPLC-ELSD): 93% (**1**), 99% (**2**), 98% (**4**), 99% (**5**), 95% (**6**), 99% (**7**), respectively.

### Cells Lines Used

Madin Darby canine kidney (MDCK) cells (Friedrich Löffler Institute, Riems, Germany), human lung carcinoma cells (A549; Institute of Molecular Virology, University of Münster, Germany), and Calu-3 cells were grown in EMEM or RPMI with 10% fetal calf serum, 2 mM l-glutamine, and 1% nonessential amino acids. Serum-free medium with trypsin (2 μg/mL for MDCK cells; 0.2 μg/mL for A549 and Calu-3 cells) was applied in the experiments.

### Influenza Viruses

Influenza viruses A/HK/1/68 (Schaper and Brümmer GmbH & Co. KG, Salzgitter, Germany), A/BLN/11/2019,^30^ A/BLN/7/2019,^30^ A/BLN/36/2019,^30^ B/NRW/33/2018,^30^ B/SN/59/2018,^30^ A/Jena/8178/09,^36^ and A/Jena/5258/09-HA-G222^37^ were used in the antiviral studies.

### Bacterial Species

Antibacterial studies were performed with *S. pneumoniae* reference strains DSM20566 (serotype 1; Leibniz Institute DSMZ-German Collection of Microorganisms and Cell Cultures, Heidelberg, Germany) and D39 (serotype 2; ZIK Septomics, Jena, Germany) as well as *S. aureus* ATCC25923 and ATCC43300 (American Type Culture Collection).

### Control Compounds

Zanamivir (GlaxoSmithKline, Brentford, UK), ampicillin (Carl-Roth GmbH, Karlsruhe, Germany), and rifampicin (Sigma-Aldrich, St. Louis, MO, USA) were used as references. Stock solutions were prepared in water (zanamivir: 10 mM; ampicillin: 10 mg/mL) or DMSO (rifampicin: 10 mg/mL).

### Validation of UPLC Quantitation Method

The quantitative method developed for extract composition was validated in terms of linearity, LOD and LOQ, precision, and accuracy. LOD and LOQ were established visually at signal-to-noise ratios of 3:1 and 10:1, respectively, by injecting solutions of known concentration. Intraday precision was studied using six replicate measurements of sample MA60 within the same day, whereas interday precision experiments were determined over 3 days using the same sample. Accuracy was assessed for compound **7** as a representative of all quantitated prenylated flavonoids. Sample MA60 was spiked with high (125%), medium (100%), and low (75%) amounts of compound **7** (Table S1, Supporting Information).

### Cytotoxicity and Anti-Influenza A Virus Activity

Cytotoxic and CPE inhibitory effects against influenza virus A/HK/1/68 (multiplicity of infection: 0.001 TCID_50_/cell) of 26 PLE extracts (10 μg/mL; triplicates) were analyzed using confluent MDCK cell monolayers, as published.^38^ Three cytotoxicity assays and two or three antiviral assays were performed. In addition, the influence of MA60, MAF, and compounds **2**, **5**, and **7** (6 half-log concentrations, maximum 100 mg/mL or 100 μM) on NADH activity and viability of confluent A549 and Calu-3 cells was evaluated using the water-soluble tetrazolium salt WST-8 (Med Chem Express, USA) and crystal violet for staining, respectively. Cells were treated at 37 °C for 72 h before adding the WST-8 solution for 2 h according to the manufacturer’s recommendations. After OD measurement at 450 vs 620 nm, the dye and dead cells were removed by three washing steps with 100 μL of phosphate-buffered saline. Thereafter, crystal violet staining was performed as published.^38^ At least three assays were performed.

### Anti-Influenza Virus Spectrum

Plaque reduction assays were performed as described previously,^38^ with several modifications. A confluent MDCK cell monolayer in 12-well plates (Greiner AG, Kremsmünster, Austria) was inoculated in each case with 0.5 mL of test medium (cell control; single) or virus suspension without (virus control; triplicate) or with serial half-log dilutions (1, 3.16, and 10 μg/mL; duplicates) of MA60, MAF, or compound **4** or **7**. After aspirating the inoculum, 1 mL of test medium containing 0.4% agar without or with the appropriate concentrations of MA60, MAF, or compound **4** or **7** was added per well, and incubation proceeded at 37 °C for 48 h (*n* = 3).

### Neuraminidase Inhibition Assay

Four hemagglutination units of influenza virus A/Jena/8178/09 (25 μL) were incubated with eight serial half-logarithmic dilutions of MA60 as indicated in Figure S3 (Supporting Information; 25 μL; duplicates) or phosphate-buffered saline (control) and a 1% erythrocyte suspension (50 μL) for 2 h at 4 °C.^39^ After obtaining the results, a further incubation at 37 °C for 24 h was performed to activate the viral neuraminidase and thus abrogate hemagglutination. The lowest compound concentration that blocked the abrogation of hemagglutination represented the minimum neuraminidase inhibitory concentration.^39^

### Antibacterial Assays

Double-concentrated half-log dilutions of reference antibiotics (ampicillin and rifampicin) and test materials (MAF, MA60, compounds **2**, **4**, and **7**) were prepared in brain-heart-infusion broth or Todd Hewith broth with yeast extract (all Carl-Roth GmbH) for *S. aureus* and *S. pneumoniae*, respectively. A volume of 50 μL of each dilution (duplicates) was dispensed into U-bottomed 96-well sterile plates (Greiner Bio-One GmbH). Then, 50 μL of bacterial suspension consisting of 2 × 10^5^ CFU/mL^40^ was inoculated to the antibiotic or test item dilutions or six wells with antibiotic-free broth for bacterial growth control. After 24 h of incubation at 37 °C and 5% CO_2_, the MIC end point was read as the lowest concentration of antibiotic or test item at which there was no visible growth (*n* = 3).

### Anti-Inflammatory Activity

To analyze the anti-inflammatory activity of MA60 (10 μg/mL), mock-treated (test medium) and MA60-treated confluent Calu-3 cell monolayers grown in 12-well plates (Greiner AG, Kremsmünster, Austria) were inoculated with test medium (cell control; duplicate) or treated with A/Jena/5258/09-HA-G222 at a multiplicity of infection (MOI) of 1 for 1 h at 37 °C. After aspirating the inoculum and washing the cells with 1 mL of PBS, 1 mL of test medium without or with MA60 (each in triplicate) was added for 24 h at 37 °C. Thereafter, total RNA was isolated with an RNeasy Mini Kit (Qiagen, Hilden). Each NP-vRNA copy number was determined after reverse transcription of 1000 ng of RNA with uni12 primer^41^ by a quantitative PCR (RT-qPCR) melted with two NP-gene targeting primers and an NP-plasmid standard. The different expression profile of cytokine mRNAs (IP10, IL-6, and IFN-β) was determined after reverse transcription of 1000 ng of RNA with oligo dT primer by semiquantitative PCR (RT-qPCR) with cytokine-targeting primers. The results were confirmed by a second experiment. PCR temperature profiles and primer sequences are shown in Tables S8–10 (Supporting Information).

### Heterocovariance Analysis

Dry weighed samples (between 2.25 and 2.4 mg) of 26 PLE extracts were dissolved in methanol-*d*_4_ to reach a concentration of 3.0 mg/mL. To avoid precipitation in the NMR tube, an aliquot of 750 μL of each extract was placed in an Eppendorf tube and centrifuged at 3000 rpm for 5 min. From the supernatants, 650 μL was transferred to NMR tubes. Standard ^1^H NMR spectra were recorded for all 26 extracts. The calculations for HetCA analyses were performed using the multiparadigm numerical computing environment MATLAB. Bioactivity data (CPE inhibitory effects against influenza virus A/HK/1/68) were taken from one experiment (*n* = 3).

### Statistical Analysis

The raw data, mean value, and standard deviation from the quantitative analysis of MDAAs, cytotoxicity, CPE inhibition, and plaque-reduction assays were analyzed using EXCEL 2016 software. IC_50_ values for inhibition of plaque production were calculated using the Four Parameter Logistic (4PL) Curve Calculator.^42^ Semiquantitative PCR results were analyzed using EXCEL 2016 software according to the method described by Pfaffl.^43^ The unpaired, two-sided Student’s *t* test was used to analyze the statistical significance of differences in Figure 5. A *p* value ≤ 0.05 was set as the cutoff for statistical significance.

## Supporting Information Available

Validation parameters for UPLC-PDA-based quantitation; parameters for extract optimization; UPLC-PDA methods used; results of quantitation; cell viability; qPCR primer sequences; temperature profiles for NP-gene quantification and cytokine analysis; heterocovariance analysis of ^1^H NMR spectra and influenza A virus inhibition data; anti-inflammatory activity, inhibition of viral neuraminidase activity (PDF)
