# Supplementary material for: 1H NMR-Based Biochemometric Analysis of Morus alba Extracts toward a Multipotent Herbal Anti-Infective
Source: J Nat Prod. 2022 Dec 21;86(1):8–17. doi: 10.1021/acs.jnatprod.2c00481 (PMC9887597; doi:10.1021/acs.jnatprod.2c00481)

Supporting Information

^{1}H NMR-based Biochemometric Analysis of *Morus alba* Extracts towards a Multipotent Herbal Anti-infective

Julia Langeder,[†,‡] Kristin Döring,[§] Hannes Schmietendorf,[§] Ulrike Grienke,[†] Michaela Schmidtke,[§,*] and Judith M. Rollinger[†,*]

[†] Division of Pharmacognosy, Department of Pharmaceutical Sciences, University of Vienna, Josef-Holaubek-Platz 2, 1090 Vienna, Austria

[‡] Vienna Doctoral School of Pharmaceutical, Nutritional and Sport Sciences, University of Vienna, Josef-Holaubek-Platz 2, 1090 Vienna, Austria

[§] Section of Experimental Virology, Department of Medical Microbiology, Jena University Hospital, Hans-Knöll-Str. 2, 07745 Jena, Germany

Table S1. Results of UPLC Method Validation.

	**1**	**2**	**3***	**4**	**5**	**6**	**7**
Levels used	9	9	n.d.	9	9	9	8
Linear range (ng/mL)	61 – 400,000	60 – 393,333	n.d.	59 – 384,333	48 – 313,333	68 – 446,667	62 – 410,000
R^2	0.99940	0.99915	n.d.	0.99945	0.99992	0.99994	0.99957
LOD (ng/mL)	40.7	6.67	n.d.	36.0	40.0	102	14.0
LOQ (ng/mL)	136	22.2	n.d.	120	133	340	46.7
Precision as standard deviation (%)							
intraday	6.6	0.9	4.2	4.8	10.1	4.2	1.2
interday	5.5	2.4	3.0	8.2	4.4	3.0	4.7
Accuracy as recovery rate (%)							
high spike	n.d.	n.d.	n.d.	n.d.	n.d.	n.d.	95.8
medium spike	n.d.	n.d.	n.d.	n.d.	n.d.	n.d.	84.2
low spike	n.d.	n.d.	n.d.	n.d.	n.d.	n.d.	86.3

n.d. … not determined

* calculated as compound **7**

Table S2. Parameters for Extract Optimization using Supercritical Fluid Extraction (SFE).

Sample name	MA organ	Applied sample (g)	Oven temperature °C	Pressure (bar)	Flow rate (mL/min)	CO_2 (mL)	Ethanol (mL)[a]	Make up - ethanol (mL)[b]	Dynamic 1 (min)[c]	Static (min)[d]	Dynamic 2 (min)[c]	Cycles	Yield (mg)	Yield %
MA01	root bark	1.00	40	300	10	7	3	0	5	5	5	1	34.10	3.4
defat step #1 of MA02	root bark	1.02	40	300	10	10	0	2	15	0	0	1	14.22	1.5
defat step #2 of MA02	root bark	1.02	40	300	10	9	1	1	15	0	0	1	n.d.	n.d.
MA02	root bark	1.02	40	300	10	7	3	0	5	5	5	1	20.34	2.0
defat step #1 of MA03	root bark	1.02	40	300	15	15	0	2	15	0	0	1	n.d.	0.0
defat step #2 of MA03	root bark	1.02	40	300	15	13.5	1.5	0.5	15	0	0	1	6.59	0.6
MA03	root bark	1.02	40	300	15	10.5	4.5	0	5	5	5	1	7.88	0.8
defat step of MA04	root bark	1.03	40	300	10	10	0	2	15	0	0	1	14.34	1.4
MA04	root bark	1.03	40	300	10	6	4	0	5	5	5	1	17.59	1.7
MA05	root bark	1.06	40	300	10	8.5	1.5	0.5	15	0	0	1	29.63	2.8
MA06	root bark	1.06	40	300	10	7	3	0	5	5	5	1	7.35	0.7
MA07	root bark	1.09	40	300	10	10	0	2	15	0	0	1	17.55	1.6
MA08	root bark	1.09	40	300	10	7	3	0	5	5	5	1	18.51	1.7
defat step of MA09	root bark	1.04	40	150	10	10	0	2	15	0	0	1	16.60	1.6
MA09	root bark	1.04	40	150	10	7	3	0	5	5	5	1	15.20	1.5
defat step of MA10	root bark	1.04	40	200	10	10	0	2	15	0	0	1	19.28	1.9
MA10	root bark	1.04	40	200	10	7	3	0	5	5	5	1	16.22	1.6
MA11	root bark	1.00	40	300	10	10	0	2	5	5	5	1	25.40	2.5
MA12	root bark	1.00	40	300	10	7	3	0	5	5	5	1	13.14	1.3
MA13	root bark	1.00	40	300	10	9.5	0.5	1.5	15	0	0	1	25.14	2.5
MA14	root bark	1.00	40	300	10	7	3	0	5	5	5	1	9.11	0.9
MA15	root bark	1.09	40	300	10	9	1	1	15	0	0	1	35.14	3.2
defat step of MA16	root bark	1.02	40	300	10	10	0	2	15	0	0	1	16.52	1.6
MA16	root bark	1.02	40	200	10	5	5	0	5	5	5	1	17.05	1.7
defat step of MA17	root bark	1.01	40	300	10	10	0	2	5	5	5	1	20.44	2.0
MA17	root bark	1.01	40	200	10	7	3	0	2	2	2	3	16.45	1.6

Sample name	MA organ	Applied sample (g)	Oven temperature °C	Pressure (bar)	Flow rate (mL/min)	CO_2 (mL)	Ethanol (mL)[a]	Make up - ethanol (mL)[b]	Dynamic 1 (min)[c]	Static (min)[d]	Dynamic 2 (min)[c]	Cycles	Yield (mg)	Yield %
defat step of MA18	root bark	1.09	40	300	10	10	0	2	5	5	5	1	20.45	1.9
MA18	root bark	1.09	40	200	10	6	4	0	5	10	5	1	20.02	1.8
defat step of MA19	root bark	1.04	50	300	10	10	0	2	15	0	0	1	20.51	2.0
MA19	root bark	1.04	50	300	10	7	3	0	5	5	5	1	17.07	1.6
MA20	root bark	1.05				ultrasonic extraction							42.69	4.1
MA21	root bark	30.0				maceration							1,600.0	5.33
MA22	root bark	5.5				reflux heating							200.0	3.64
MA23	root bark	4.7				reflux heating							400.0	8.51
MA24	root bark	5.48				decoction							262.2	4.78
MA25	leaves	1.00	40	300	10	7	3	0	5	5	5	1	45.96	4.6
MA26	leaves	1.04	40	300	10	10	0	2	15	0	0	1	5.43	0.6
MA27	leaves	1.00	40	300	10	7	3	0	5	5	5	1	28.52	2.9
MA28	leaves	1.03				ultrasonic extraction							48.92	5.0
MA29	twigs	1.04	40	300	10	7	3	0	5	5	5	1	29.59	3.1
MA30	twigs	1.04	40	300	10	10	0	2	15	0	0	1	5.23	0.5
MA31	twigs	1.00	40	300	10	7	3	0	5	5	5	1	16.62	1.7
defat step of MA32	twigs	1.03	40	300	10	9.5	0.5	1.5	15	0	0	1	6.90	0.7
MA32	twigs	1.00	40	300	10	7	3	0	5	5	5	1	13.76	1.4
MA33	twigs	1.00				ultrasonic extraction							28.10	2.8
MA34	fruits	1.03	40	300	10	7	3	0	5	5	5	1	215.33	22.2
MA35	fruits	1.00	40	300	10	10	0	2	15	0	0	1	24.05	2.4
MA36	fruits	1.00	40	300	10	7	3	0	5	5	5	1	139.02	13.9
MA37	fruits	1.00				ultrasonic extraction							456.55	45.7

[a] Added amount of ethanol as co-solvent to the supercritical CO_2 to obtain the super-/subcritical solvent mixture (10 mL in total)
[b] Added amount of ethanol after passage of the backpressure regulator, when the CO_2 turns into a gas again to keep extractives in solution
[c] Extraction setting, where continuously new solvent is pumped through the sample
[d] Extraction setting, where the sample is soaked with solvent and no new solvent is pumped through the sample

Table S3. Results from the Quantitation of SFE Extracts and Traditional Extracts from *M. alba* Root Bark.

sample	**1** [%]	± SD [%]	**2** [%]	± SD [%]	**3** [%]	± SD [%]	**4** [%]	± SD [%]	**5** [%]	± SD [%]	**6** [%]	± SD [%]	**7** [%]	± SD [%]	**Total sanggenon content [%]**
MA01	1.43	0.03	1.22	0.05	0.09	0.13	0.05	-	n.f.	-	0.15	0.01	1.19	0.09	**4.12**
MA02	1.51	0.02	1.49	0.03	n.f.	-	n.f.	-	0.08	0.01	0.19	-	1.42	0.04	**4.66**
MA03	n.d.	-	n.d.	-	n.d.	-	n.d.	-	n.d.	-	n.d.	-	n.d.	-	**n.d.**
MA04	1.70	0.28	1.91	-	n.f.	-	n.f.	-	0.08	-	0.18	-	1.99	0.04	**5.87**
MA05	1.03	0.01	0.80	0.06	n.f.	-	n.f.	-	0.11	0.01	0.19	0.05	1.71	0.09	**3.85**
MA06	n.d.	-	n.d.	-	n.d.	-	n.d.	-	n.d.	-	n.d.	-	n.d.	-	**n.d.**
MA07	n.d.	-	n.d.	-	n.d.	-	n.d.	-	n.d.	-	n.d.	-	n.d.	-	**n.d.**
MA08	3.02	0.62	4.30	0.16	0.26	0.01	0.11	0.01	0.10	-	0.27	-	3.49	0.05	**11.54**
MA09	3.61	0.75	5.17	0.59	0.57	0.15	0.28	0.04	0.16	0.01	0.35	0.01	4.24	0.02	**14.39**
MA10	3.91	0.61	5.98	0.44	0.43	0.01	0.21	0.01	0.12	0.01	0.30	0.03	6.17	0.03	**17.12**
MA11	n.f.	-	n.f.	-	n.f.	-	n.f.	-	n.f.	-	n.f.	-	n.f.	-	**n.f.**
MA12	1.93	0.05	2.91	0.11	0.05	0.01	0.07	0.01	0.09	-	0.21	0.02	3.24	0.02	**8.51**
MA13	1.00	-	1.04	0.03	0.15	0.01	n.f.	-	0.10	-	n.f.	-	1.38	0.05	**3.66**
MA14	2.63	0.22	3.80	0.10	n.f.	-	0.18	0.02	0.11	-	0.26	0.02	2.88	0.04	**9.85**
MA15	1.14	0.02	1.59	0.06	0.05	0.01	0.05	-	0.07	-	0.18	0.01	1.49	0.08	**4.56**
MA16	4.20	0.83	6.06	0.88	0.64	0.21	0.34	0.11	0.21	0.07	0.33	0.01	4.37	0.04	**16.15**
MA17	4.42	0.83	7.37	0.89	0.83	0.23	0.55	0.12	0.25	0.08	0.46	0.01	6.01	0.08	**19.89**
MA18	3.87	0.26	5.17	0.63	0.51	0.21	0.38	0.13	0.19	0.08	0.36	0.01	4.08	0.11	**14.56**
MA19	3.22	0.02	5.78	0.06	0.28	0.01	0.27	0.02	0.15	0.02	0.34	0.07	6.69	0.16	**16.73**
MA20	1.53	0.03	2.05	0.02	n.f.	-	0.04	0.01	n.f.	-	0.18	0.01	1.50	0.06	**5.31**
MA21	0.21	0.01	1.06	0.01	0.05	0.00	0.01	0.00	0.04	0.00	0.18	0.00	1.04	0.01	**2.59**
MA22	1.45	0.04	1.63	0.02	0.09	0.00	0.47	0.03	0.16	0.01	0.40	0.0	1.01	0.02	**5.21**
MA23	0.68	0.01	1.67	0.16	0.03	0.02	0.23	0.04	0.31	0.02	0.42	0.05	1.13	0.08	**4.47**
MA24	n.f.	-	n.f.	-	n.f.	-	n.f.	-	n.f.	-	n.f.	-	n.f.	-	**n.f**

Table S4. Parameters for Extract Optimization using Pressurized Liquid Extration (PLE).

Sample name	MA organ	Applied sample (g)	Solvent (ratio in brackets)	Temperature °C	Method mode	Static (min)	Cycles	Rinse %	Purge (s)	Yield (mg)	Yield %
MA38	root bark	1.77	ethanol	50	standard	5	2	60	100	57.28	3.24
defat step of MA39	root bark	1.23	n-hexane	50	standard	5	1	60	100	n.d.	n.d.
MA39	root bark	1.23	ethanol/water	50	standard	5	1	60	100	108.47	8.82
defat step of MA40	root bark	1.4	n-hexane	50	standard	5	2	60	100	n.d.	n.d.
MA40	root bark	1.4	ethanol	50	standard	5	2	60	100	22.76	1.63
defat step of MA41	root bark	1.98	n-hexane	100	fixed volume	5	n.d.	0	100	n.d.	n.d.
MA41	root bark	1.98	ethanol	100	fixed volume	5	n.d.	0	100	43.27	2.19
defat step of MA42	root bark	2.11	n-hexane	100	pressure	5	n.d.	0	100	n.d.	n.d.
MA42	root bark	2.11	ethanol	100	pressure	5	n.d.	0	100	60.63	2.87
defat step of MA43	root bark	2.12	n-hexane	100	flow	15	1	0	100	n.d.	n.d.
MA43	root bark	2.12	ethanol	100	flow	15	1	0	100	109.58	5.17
defat step of MA44	root bark	2.35	n-hexane	100	flow	5	1	0	100	n.d.	n.d.
MA44	root bark	2.35	acetone	100	flow	5	1	0	100	36.41	1.55
defat step of MA45	root bark	2.47	n-hexane	100	flow	15	1	0	100	n.d.	n.d.
MA45	root bark	2.47	ethanol	100	flow	5	1	0	100	92.05	3.73
MA46	root bark	2.43	dichloromethane	100	flow	5	1	0	100	65.79	2.71
MA47	root bark	2.43	dichloromethane(5)-methanol(1)	100	flow	5	1	0	100	33.48	1.38
MA48	root bark	2.43	dichloromethane(5)-methanol(2)	100	flow	5	1	0	100	19.42	0.80
cycle #2 of MA48	root bark	2.43	dichloromethane(5)-methanol(3)	100	flow	5	1	0	100	13.06	0.54
cycle #3 of MA48	root bark	2.43	dichloromethane(1)-methanol(1)	100	flow	5	1	0	100	13.64	0.56
MA49	root bark	2.43	methanol	100	flow	5	1	0	100	21.15	0.87
defat step of MA50	root bark	2.24	n-hexane	100	flow	5	1	0	100	n.d.	n.d.
MA50	root bark	2.24	isopropanol	100	flow	5	1	0	100	35.09	1.57
defat step of MA51	root bark	2.21	n-hexane	100	flow	5	1	0	100	n.d.	n.d.

Sample name	MA organ	Applied sample (g)	Solvent (ratio in brackets)	Temperature °C	Method mode	Static (min)	Cycles	Rinse %	Purge (s)	Yield (mg)	Yield %
MA51	root bark	2.21	acetonitrile	100	flow	5	1	0	100	38.88	1.76
MA52	root bark	2.44	n-hexane	100	flow	5	1	0	100	56.52	2.32
defat step of MA53	root bark	2.44	ethanol	100	flow	5	1	0	100	103.47	4.24
MA53	root bark	2.49	petroleum ether	100	flow	20	1	0	100	64.88	2.61
MA54	root bark	2.49	isopropanol	100	flow	20	1	0	100	39.66	1.59
defat step of MA55	root bark	2.51	petroleum ether	120	flow	5	1	0	100	60.46	2.41
MA55	root bark	2.51	isopropanol	120	flow	5	1	0	100	37.75	1.50
defat step of MA56	root bark	2.55	petroleum ether	100	flow	5	1	0	100	56.58	2.22
MA56	root bark	2.55	isopropanol	80	flow	5	1	0	100	32.65	1.28
defat step of MA57	root bark	2.47	petroleum ether	100	flow	5	1	0	100	n.d.	n.d.
MA57	root bark	2.47	petroleum ether(1)-isopropanol(1)	100	flow	5	1	0	100	25.4	1.03
defat step of MA58	root bark	2.26	petroleum ether	100	flow	5	1	0	100	n.d.	n.d.
MA58	root bark	2.26	ethanol(1)-isopropanol(1)	100	flow	5	1	0	100	62.43	2.76
defat step of MA59	root bark	2.44	n-hexane	120	flow	5	1	0	100	n.d.	n.d.
MA59	root bark	2.44	petroleum ether(1)-isopropanol(1)	80	flow	5	1	0	100	22.06	0.90
defat step of MA60	root bark	2.27	n-hexane	120	flow	5	1	0	100	n.d.	n.d.
MA60	root bark	2.27	petroleum ether(1)-isopropanol(2)	80	flow	5	1	0	100	23.15	1.02
defat step of MA61	root bark	2.36	n-hexane	120	flow	5	1	0	100	n.d.	n.d.
MA61	root bark	2.36	petroleum ether(2)-isopropanol(1)	80	flow	5	1	0	100	18.83	0.80
defat step of MA62	root bark	2.27	n-hexane	120	flow	5	1	0	100	n.d.	n.d.
MA62	root bark	2.27	petroleum ether(1)-isopropanol(1)	60	flow	5	1	0	100	17.36	0.76
defat step of MA63	root bark	2.39	n-hexane	120	flow	5	1	0	100	n.d.	n.d.
MA63	root bark	2.39	petroleum ether(1)-1-propanol(1)	80	flow	5	1	0	100	23.77	0.99

Table S5. Results from the Quantitation of 33 PLE Extracts from *M. alba* Root Bark.

Sample	**1** [%]	± SD [%]	**2** [%]	± SD [%]	**3** [%]	± SD [%]	**4** [%]	± SD [%]	**5** [%]	± SD [%]	**6** [%]	± SD [%]	**7** [%]	± SD [%]	**Total sanggenon content [%]**
MA38	1.80	0.40	1.84	0.22	0.04	0.01	n.f.	-	0.08	0.01	0.21	0.01	1.90	0.06	**5.87**
MA39	0.89	0.01	1.59	0.03	n.f.	-	0.04	0.01	0.08	0.01	0.31	0.01	1.52	0.02	**4.45**
MA40	4.54	0.09	6.63	0.07	0.47	0.01	0.45	0.01	0.13	-	0.47	0.02	4.68	0.05	**17.37**
MA41	2.46	0.41	3.84	0.16	0.12	-	0.15	0.02	0.18	0.01	0.34	-	2.74	0.01	**9.83**
MA42	2.23	0.03	4.02	0.05	0.16	-	0.25	0.04	0.17	-	0.35	0.02	2.87	0.05	**10.05**
MA43	1.28	0.09	2.21	0.04	n.f.	-	0.07	-	0.11	-	0.29	0.02	1.67	0.08	**5.63**
MA44	6.48	0.35	11.23	0.65	1.17	0.09	0.96	0.05	0.25	0.02	0.71	0.03	7.56	0.50	**28.34**
MA45	1.53	0.17	3.19	0.03	0.04	-	0.19	0.05	0.15	0.01	0.38	0.01	2.44	0.05	**7.92**
MA46	1.27	0.02	0.28	0.02	0.13	-	n.f.	-	0.12	0.02	0.27	0.02	1.22	0.04	**3.29**
MA47	2.57	0.02	5.42	0.04	n.f.	-	0.42	0.01	0.13	-	0.22	-	2.80	0.01	**11.55**
MA48	n.f.	-	n.f.	-	n.f.	-	n.f.	-	n.f.	-	n.f.	-	n.f.	-	**n.f.**
MA49	n.f.	-	n.f.	-	n.f.	-	n.f.	-	n.f.	-	n.f.	-	n.f.	-	**n.f.**
MA50	5.13	0.05	8.97	0.14	0.99	0.07	0.54	0.08	0.27	0.07	0.53	0.01	6.07	0.06	**22.50**
MA51	4.19	0.38	8.12	0.65	0.77	0.23	0.62	0.12	0.27	0.06	0.62	0.01	5.89	0.07	**20.49**
MA52	n.f.	-	n.f.	-	n.f.	-	n.f.	-	n.f.	-	n.f.	-	n.f.	-	**n.f.**
MA53	n.f.	-	n.f.	-	n.f.	-	n.f.	-	n.f.	-	n.f.	-	n.f.	-	**n.f.**
MA54	5.45	0.10	10.72	0.15	0.76	0.01	0.90	0.01	0.33	-	0.82	0.01	8.10	0.04	**27.08**
MA55	5.03	0.04	10.58	0.12	1.13	0.07	1.13	0.11	0.53	0.13	1.01	0.16	7.07	0.04	**26.47**
MA56	5.92	0.49	11.48	0.91	1.17	0.34	0.85	0.18	0.28	0.07	0.73	0.05	8.24	0.12	**28.67**
MA57	5.31	0.01	9.48	0.10	0.80	-	0.66	-	0.26	0.01	0.68	0.02	7.77	0.09	**24.97**
MA58	2.70	0.03	4.56	0.02	0.21	-	0.29	0.01	0.12	0.01	0.42	0.01	3.47	0.02	**11.76**
MA59	6.72	0.05	11.62	0.03	1.56	0.06	0.99	0.16	0.34	0.07	0.70	0.03	8.72	0.10	**30.65**
MA60	7.17	0.04	10.68	0.10	1.24	0.04	1.20	0.05	0.82	0.04	0.96	0.06	6.87	0.10	**28.95**
MA61	6.35	0.35	11.78	0.67	1.44	0.33	1.09	0.13	0.33	0.05	0.85	0.05	9.71	0.05	**31.54**
MA62	5.89	0.70	8.67	0.72	1.07	0.30	1.09	0.18	0.30	0.07	0.65	-	7.23	0.04	**24.88**
MA63	5.55	0.19	9.16	0.16	0.84	0.02	0.75	0.02	0.30	0.01	0.79	0.03	7.83	0.20	**25.23**

Table S6. Results from cytotoxicity studies in two lung epithelial cell lines.[a]

Extract/ Compound	Concentration	reducing cell viability by 50%[a]				reducing NADH activity by 50%[a]			
		A549 cells		Calu-3 cells		A549 cells		Calu-3 cells	
		Mean	SD	Mean	SD	Mean	SD	Mean	SD
**MAF**	µg/ml	17.42	2.20	24.67	8.41	29.23	3.49	25.65	10.36
**MA60**	µg/ml	37.36	7.64	30.30	12.52	83.03	7.96	29.12	6.16
**1**	µM	48.62	8.52	62.79	32.91	63.22	15.76	66.79	21.46
**2**	µM	58.07	16.29	49.59	23.75	87.24	2.13	37.58	14.77
**4**	µM	12.51	0.30	19.88	5.18	21.50	3.01	21.06	6.23
**7**	µM	12.53	0.41	16.13	5.08	19.52	0.83	13.63	2.60

[a] Three independent assays were performed to determine the mean concentrations with standard deviation (SD) reducing NADH activity and cell viability by 50%, respectively.

Table S7. UPLC Conditions used for Separation.

Sample	Column	Flow Rate (mL/min)	Solvent Gradients			Obtained Fraction/Compound
MAM	PURIFLASH COLUMN 25 SILICA HC 200G 25μg (20bar)	100	Time (min)	%A (DCM)	%B (MeOH)	MAM01_01-MAM01_11
			00:00	100	0	
			26:54	90	10	
			01:48:00	60	40	
MAM01_04	GEMINI-NX C_{18} (5 μm, 110Å, 250×10 mm) Phenomenex®	5	Time (min)	%A (Water)	%B (ACN)	Sanggenon E (**6**)
			00:00	95	05	
			05:00	57	43	
			20:00	55	45	
			50:00	54	46	
			55:00	02	98	
			01:00:00	02	98	
MAM01_05	GEMINI-NX C_{18} (5 μm, 110Å, 250×10 mm) Phenomenex®	5	Time (min)	%A (Water)	%B (ACN)	Sanggenon C (**7**) Sanggenon O (**5**)
			00:00	95	05	
			05:00	60	40	
			01:05:00	52	48	
			01:10:00	02	98	
			01:30:00	02	98	
MAM01_06	GEMINI-NX C_{18}	5	Time (min)	%A (Water)	%B (ACN)	Sanggenon D (**2**)
			00:00	95	05	

	(5 µm, 110Å,		05:00	60	40	
	250×10 mm)		30:00	55	45	
	Phenomenex®		35:00	00	100	
			45:00	00	100	
MAF	GEMINI-NX	5	Time	%A	%B	Sanggenon G (**4**)
	C_{18}		(min)	(Water)	(ACN)	
	(5 µm, 110Å,					
	250×10 mm)		00:00	95	05	
	Phenomenex®		05:00	60	40	
			01:05:00	52	48	
			01:10:00	02	98	
			01:30:00	02	98	
MA60LS2	GEMINI-NX	5	Time	%A	%B	Kuwanon L (**1**)
	C_{18}		(min)	(Water)	(ACN)	
	(5 µm, 110Å,					
	250×10 mm)		00:00	95	05	
	Phenomenex®		05:00	60	40	
			30:00	55	45	
			35:00	02	98	
			45:00	02	98	

Table S8. qPCR Primer Sequences.

NP_forward	5′-GCA TCT GTC GGA AGA ATG AT-3′
NP_reverse	5′-AAG CAC CAT CCT CTC TAT TG-3′
GAPDH_forward	5′-CTC TGC TCC TCC TGT TCG AC-3′
GAPDH_reverse	5′-CAA TAC GAC CAA ATC CGT TG-3′
IP-10_forward	5′-CCA GAA TCG AAG GCC ATC AA-3′
IP-10_reverse	5′-TTT CCT TGC TAA CTG CTT TC-3′
IL-6_forward	5′-CAG CCC TGA GAA AGG AGA CATG-3′
IL-6_reverse	5′-GCA TCC ATC TTT TTC AGC CATC-3′
IFN-β_forward	5′-TCT GGC ACA ACA GGT AGT AG-3′
IFN-β_reverse	5′-GAG AAG CAC AAC AGG AGA GC-3′

Table S9. Temperature Profile for NP-Gene Quantification.

95° C	5 min	
95° C	10 sec	37 cycles
55° C	30 sec	
72° C	120 sec	
60° C – 95° C	Melting curve analysis	
20° C	10 sec	

Table S10. Temperature Profile for Cytokine Analysis.

95° C	5 min	
95° C	10 sec	35 cycles
59° C	30 sec	
72° C	120 sec	
60° C – 95° C	Melting curve analysis	
20° C	10 sec	

Figure S1. NMR Pseudo-Spectrum Showing the Heterocovariance Analysis (HetCA) of ^{1}H NMR Spectra and Influenza A Virus Inhibition Data of 26 Extracts Produced by PLE. Below, ^{1}H NMR spectra of isolates are given between (A) δ = 0 ppm and 9 ppm, and (B) zoomed in between δ = 5 ppm and 8.5 ppm for comparative reasons.

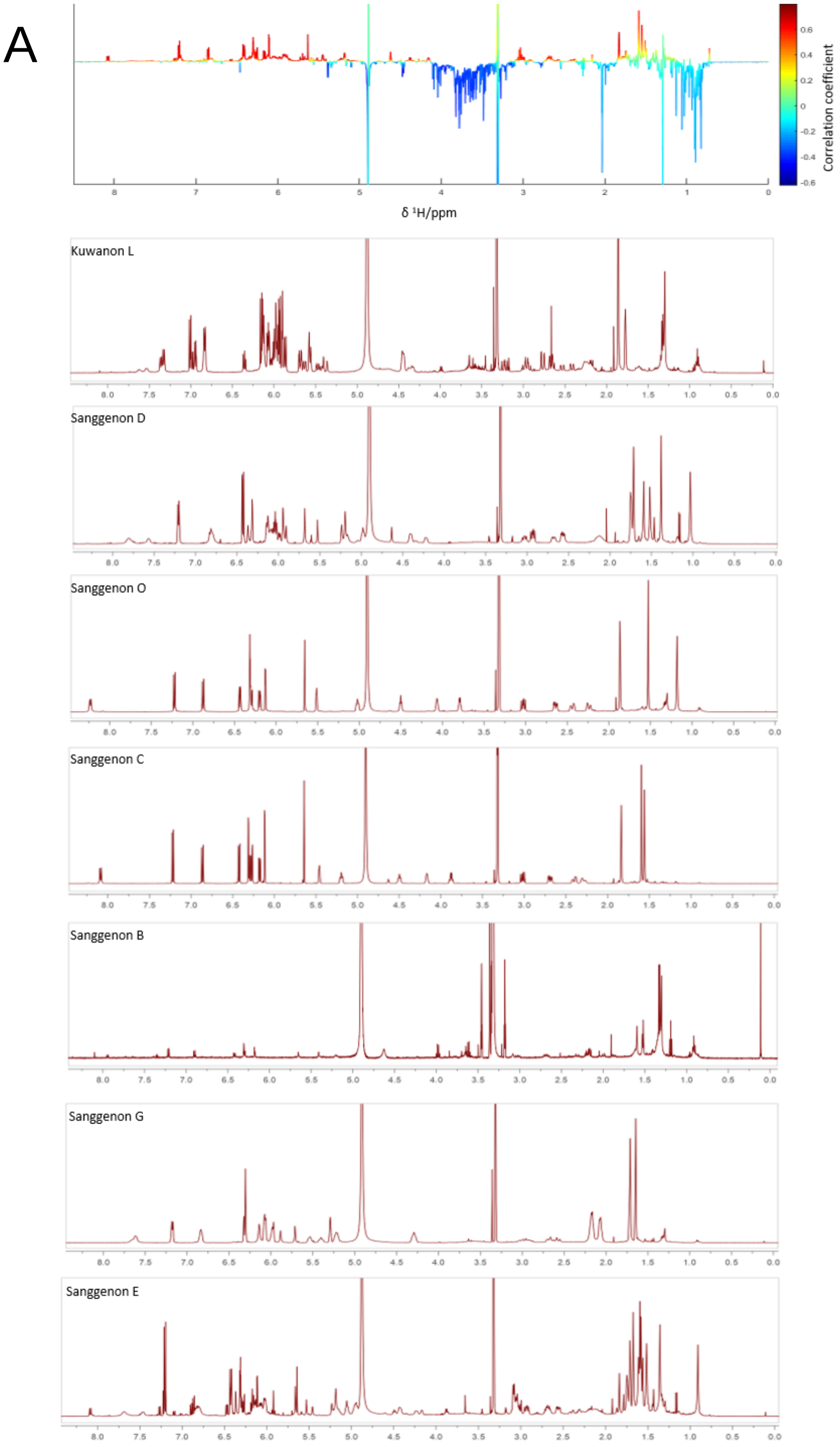

B

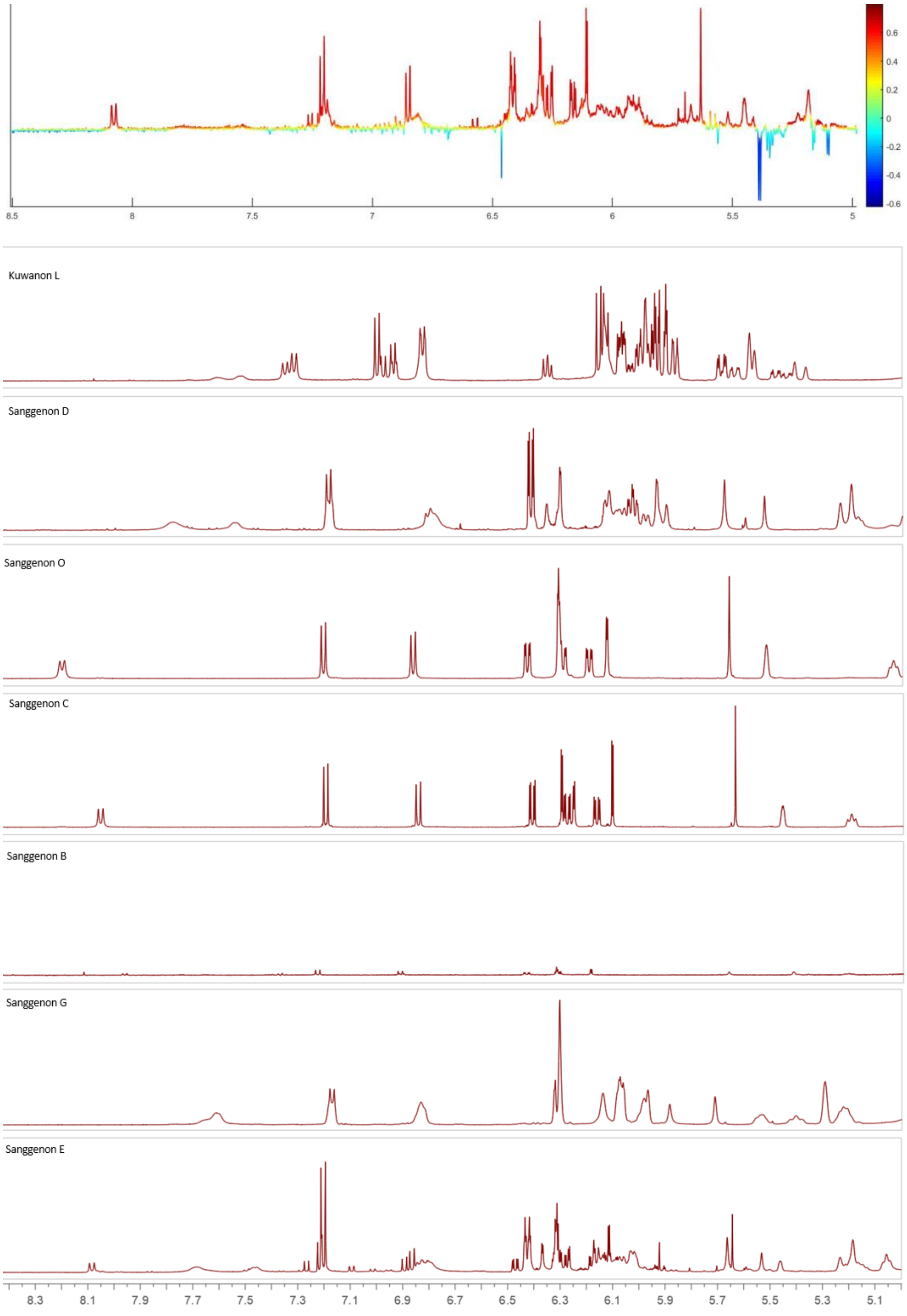

0.6
0.4
0.2
0
-0.2
-0.4
-0.6
8.5
8
7.5
7
6.5
6
5.5
5
Kuwanon L
Sanggenon D
Sanggenon O
Sanggenon C
Sanggenon B
Sanggenon G
Sanggenon E
8.3
8.1
7.9
7.7
7.5
7.3
7.1
6.9
6.7
6.5
6.3
6.1
5.9
5.7
5.5
5.3
5.1

Figure S2. Results from studies on the effect of MA60 (10 µg/mL) on viral RNA synthesis (A) and virus-induced cytokine mRNA induction (B) in Calu-3 cells. *p<0.05, **p<0.01 in unpaired two-sided Student´s t-test.

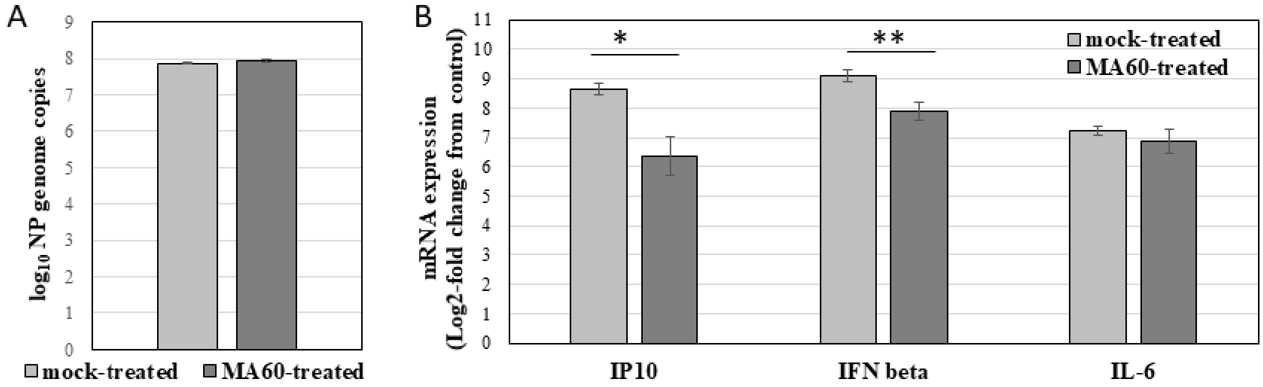

Figure S3. Results from studies on the effect of MA60 (A) on the influenza virus-induced hemagglutination of human erythrocytes and (B) viral neuraminidase activity.

(A) Phosphate-buffered saline (Co 1: 8178/09: -) or four hemagglutination units of influenza virus A/Jena/8178/09 (8178/09) (Co 2: 8178/09: +) were mixed with phosphate-buffered saline or with serial half-logarithmic dilutions of MAF and and MA60 (10 to 0.003 µg/mL). A human erythrocyte solution was added before incubating the mixture at 4 °C for 2 h. (B) After protocolling the effect on hemagglutination, we further incubated the test at 37 °C overnight allowing activation of viral neuraminidase activity. Three experiments were performed. *The results for control and zanamivir were recently published (Döring et al. 2021)

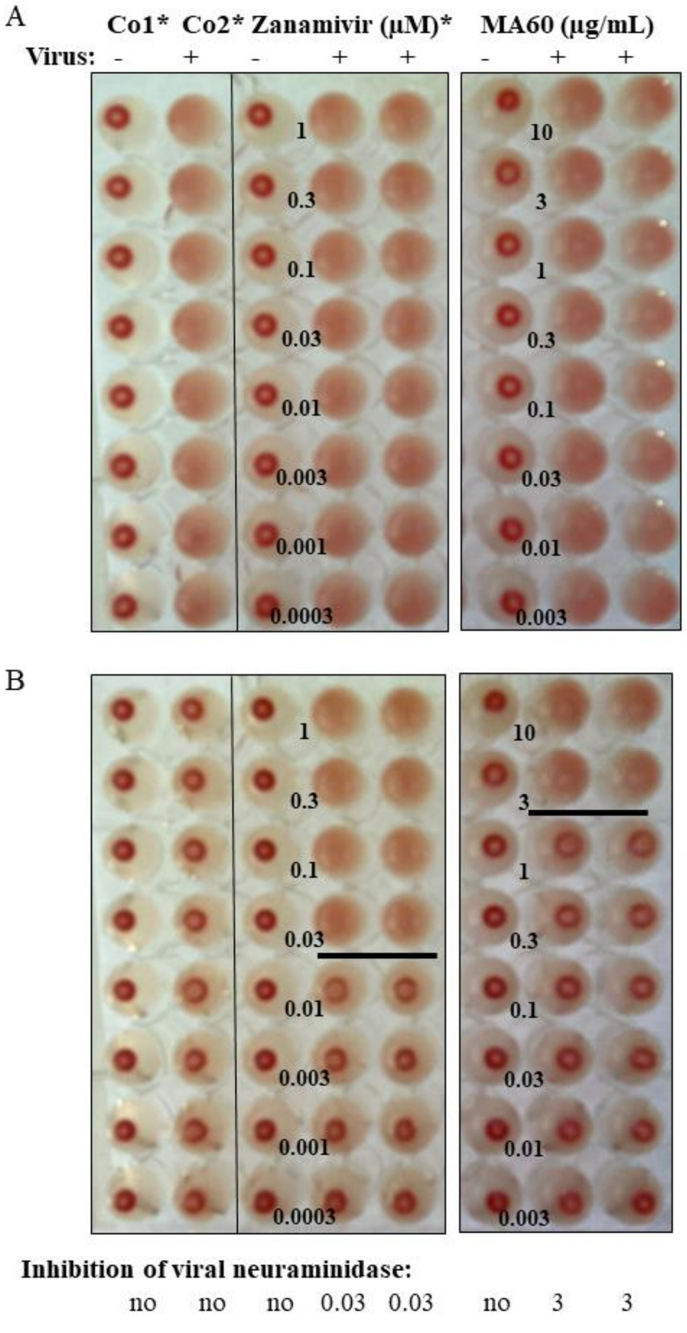

Supplement: Supplementary file 1 — np2c00481_si_001.pdf [file np2c00481_si_001.pdf]
